# Screening tool development for hand surgery referrals in systemic sclerosis

**DOI:** 10.1016/j.clinsp.2023.100270

**Published:** 2023-08-17

**Authors:** Marcos Felipe Marcatto de Abreu, Síbila Landin, Fernanda Yuri Yuamoto, Carolina Lins, Eduardo Paiva Magalhães, Maurício Etchebehere

**Affiliations:** a Department of Orthopedics, Rheumatology and Traumatology, Faculdade de Ciências Médicas, Universidade Estadual de Campinas (UNICAMP), Campinas, São Paulo, Brazil; b Department of Therapeutic Processes, Catholic University of Temuco, Temuco, Araucanía, Chile

**Keywords:** Scleroderma, systemic, Orthopedic procedures, Cross-sectional studies, Skin ulcer, Referral and consultation

## Abstract

**Background:**

Systemic Sclerosis (SSc) patients may need hand surgery.

**Objective:**

To develop a screening tool for rheumatologists to identify potential candidates with systemic sclerosis for hand surgery, optimizing referrals.

**Methods:**

A pilot cross-sectional study from January 2015 to December 2016. Sample size: 51 participants. Inclusion criteria: ≥ 18 years old, meeting the 2013 American College of Rheumatology/European League Against Rheumatism (ACR/EULAR) classification criteria for SSc and hand impairment. Data collected: age, sex, race, disease duration, SSc subtypes, vasodilator use, skin thickness, finger stiffness, presence of Digital Ulcers (DU) and/or calcinosis, presence of Raynaud's Phenomenon (RP) attacks, health status and disability, disease status, pain intensity and functional status of the hands. Data were analyzed by a multivariate logistic regression model.

**Results:**

Fulfillment of surgical criteria: 68.8%. The surgical group had higher scores on the HAQ-DI (1.39 vs. 0.96, *p =* 0.032) and CHFS (25.0 vs. 12.0, *p =* 0.005) questionnaires, and a higher frequency of DU (91.43% vs. 18.75%, *p <* 0.0010), calcinosis (60.0% vs. 0.0%, *p <* 0.001), use of vasodilators (100.0% vs. 75.0%, *p =* 0.007) and digital stiffness (28.57% vs. 0.0%, *p =* 0.017). The presence of DU increased the chance of surgical indication by 46.2 times (ORIC 95% = 8.23 to 259.49). The statistical model showed good accuracy (86.3%, *p <* 0.001), sensitivity (91.4%), and specificity (81.2%).

**Conclusion:**

The presence of DU in SSc could be used as a screening feature for early identification and referral of potential candidates for hand surgery.

## Highlights

• Identification of potential candidates for hand surgery in Systemic Sclerosis.
• Presence of digital ulcers increases the likelihood of surgical indication.
• Development of a predictive tool to identify candidates for surgical treatment.

## Introduction

Communication between physicians and hand surgeons is essential in the multidisciplinary approach of SSc patients. Timely referral to a hand surgery specialist can ensure that those with hand manifestations receive the most appropriate treatment as they tend to be in better general and hand conditions should a surgical procedure be required. On the other hand, late referrals can lead to less satisfactory results, both due to the greater involvement of the hand and the more deteriorated clinical conditions of these individuals.^1^ Although there is no established perfect “Window” for referral to be made, early identification of potential surgical candidates could optimize surgical outcomes and the quality of life for these individuals.

Systemic sclerosis is a rare multisystem disease caused by immune-mediated endothelial dysfunction, characterized by tissue ischemia and fibrosis.^2^, ^3^, ^4^ Hand manifestations include skin thickening, joint contractures, calcium deposits, Raynaud's Phenomenon (RP) attacks, nervous compressive symptoms, pain, digital gangrene, and bone infection, all of which contribute to disease-related disability and disfigurement.^3^ The treatment of these alterations is mainly conservative, based on the use of immunosuppressive and anti-inflammatory medications, vasodilators, prostacyclin, and phosphodiesterase-5 inhibitors, rehabilitation and education programs.^5^^,^^6^ However, in certain scenarios when non-surgical treatments have failed, selected surgeries can help relieve pain, improve function, and provide better cosmesis.^6^ Among possible surgical procedures are listed: arterial sympathectomies ‒ to improve hand perfusion ‒ arthrodesis of interphalangeal and wrist joints, and resection arthroplasties of the metacarpophalangeal joint and the trapezius ‒ to improve hand position and function ‒ calcinosis resection ‒ for pain relief ‒ nerve decompressions, and fingertip amputations ‒ for intractable ulcers or bone infection.^1^^,^^7^ However, despite its frequent involvement, general practitioners and rheumatologists often neglect the treatment of hand manifestations and their surgical solutions. This is related not only to the general health problems of the patient but also to insufficient knowledge and training of the doctors in the available surgical options.^8^^,^^9^ Some clinical signs and assessment tools have been applied to disease monitoring and evaluation of therapeutic intervention results.^10^ However, none of them has been used to assist in referral to a hand surgery specialist.

Therefore, given the low rates of timely referrals to the hand surgeon, we carried out a pilot study to develop and propose a practical tool that could assist in the early identification and referral of those individuals who could benefit from hand surgery, making surgical referrals part of a more evidence-based approach.

## Methods

### Study design

The study was approved by the local research ethics committee (protocol number: 23261013.8.0000.5404).

We performed a cross-sectional evaluation of individuals registered at the scleroderma unit of our institution (Hospital de Clínicas of the State University of Campinas – HC UNICAMP) between January 2015 and December 2016.

### Setting

We obtained a convenience sample of individuals with SSc during their routine consultation at the scleroderma unit at HC-UNICAMP.

### Participants

Potential participants were approached while in the waiting room or after their appointment. After signing the informed consent, the individuals were surveyed by a hand surgeon and a hand therapist.

Only individuals over 18 years of age, fulfilling the 2013 ACR/EULAR classification criteria for SSc,^11^ and without a previous hand operation were included in the study. Those with other forms of scleroderma and unwilling to participate were excluded.

### Data collection

The survey instruments captured participants’ demographic, disease, and hand function information. The demographic data collected were age, sex, and race. Information about disease duration, SSc subtype,^12^ vasodilator use, RP attacks in the previous week, skin thickness (mRSS),^13^ finger motion (delta FTP distance),^14^ digital ulcer and calcinosis presence, digital gangrene or infection, pain intensity (VAS),^15^ forearm limitation, and carpal tunnel symptoms were also collected. Finally, participants answered the HAQ-DI, SHAQ,^10^ and CHFS^16^ questionnaires. The questionnaires used are validated for the Portuguese language.

The surgical indications and the corresponding proposed procedures were as follows:

a) Ischemia (digital ischemia accompanied by severe pain, ulceration, or infection) ‒ sympathectomy or amputation.
b) Calcinosis (calcium deposits associated with extrusion, severe pain, or infection) ‒ excision of calcium deposits.
c) Contracture (finger, wrist, and first web contraction associated with ulceration due to skin break, and poor finger motion) ‒ arthrodesis of the proximal interphalangeal joint or wrist, arthroplasty of the metacarpophalangeal joint, trapezium resection.
d) Carpal tunnel syndrome (carpal tunnel symptoms unresponsive to conservative treatment) ‒ carpal tunnel release.^1^^,^^17^, ^18^, ^19^, ^20^, ^21^

### Data analysis

For statistical purposes, we considered poor finger movement for delta-FTP values less than 40 mm^14^^,^^16^ and severe pain for values greater than 7 in the VAS.^22^

Only one event (indication for surgical treatment) was considered for each person. Participants were grouped according to eligibility for surgical treatment.

We calculated descriptive statistics for participant characteristics. The Shapiro–Wilk test was used to verify data distribution and the Levene test for the homogeneity of variances. Comparisons between continuous variables were done using *t* Student and Mann–Whitney *U* tests and between categorical variables using the Chi-Square and Fisher's exact tests, as appropriate. Those variables demonstrating significant statistical differences between the groups were included in a stepwise multivariate binary regression model. The Hosmer–Lemeshow test was used to verify the model's goodness of fit, and the Wald test to analyze the significance of coefficients in the model. The strength of the association between each variable in the model was presented using an Odds Ratio (OR) with a Confidence Level of 95% (95% CI). We constructed Receiver Operating Characteristic (ROC) curves from the values predicted by the logistic regression model and analyzed the Area Under the Curve (AUC). Sensitivity and specificity tests were used to verify the accuracy of the model. The AUC was considered “excellent” (0.9 ≤ AUC < 1), “good” (0.8 ≤ AUC < 0.9), “fair” (0.7 ≤ AUC < 0.8), or “poor” (AUC < 0.7). All analyzes were performed using PASW statistics 18.0 software (Statistical Package for the Social Sciences Inc. Chicago, USA), with a significance level (α) of 5% (*p <* 0.05).

## Results

### Participants

Fifty-four subjects were selected and 51 (27‒76 years old) were included in the study. Three individuals were excluded for not meeting the 2013 ACR/EULAR classification criteria for SSc.

### Descriptive and outcome data

Women represented 92.2% of the sample size, and the mean age of the participants was 50 years old (27‒76). Regarding racial division, 65% declared themselves as white, 27.5% as brown, and 7.5% as black.

Established surgical criteria were met by 68.8% of the participants. These individuals presented higher scores on HAQ-DI (mean score = 1.39 vs. 0.96, *p =* 0.032), and CHFS (median score = 25.0 vs. 12.0, *p =* 0.005) questionnaires. They also presented higher frequency of DU (91.43% vs. 18.75%, *p <* 0.0010), calcinosis (60.0% vs 0.0%, *p <* 0.001), use of vasodilators (100.0% vs. 75.0%, *p =* 0.007), and digital stiffness (28.57% vs. 0.0%, *p =* 0.017) (Table 1).

**Table 1:** Participants' characteristics.

Variables	Total (*n* = 51)	Surgical Indication		*p*-value
		Yes (*n* = 35)	No (*n* = 16)	
Age^a^ (years)	50.0 ± 12.0	49.9 ± 12.7	50.0 ± 10.7	0.988^ST^
Time since diagnosis^b^ (years)	8.0 [4.0‒16.0]	7.0 [4.0‒11.8]	8.0 [4.0‒16.0]	0.597^MW^
Rodnan^a^	23.2 ± 8.9	24.2 ± 9.1	20.9 ± 8.3	0.229^ST^
HAQ-DI^a^ (score)	1.25 ± 0.67	1.39 ± 0.67	0.96 ± 0.58	0.032^ST^
SHAQ^a^ (score)	1.06 ± 0.56	1.16 ± 0.58	0.86 ± 0.45	0.073^ST^
CHFS^b^ (score)	18.0 [9.0‒38.0]	25.0 [10.0‒46.0]	12.0 [5.0‒17.0]	0.005^MW^
Gender				
Male	7.8% (44/51)	3 (8.57%)	1 (6.25%)	1.000^FE^
Female	92.2% (47/51)	32 (91.43%)	15 (93.75%)	
Systemic sclerosis				
Limited	62.7% (32/51)	21 (60.00%)	11 (68.75%)	0.549^CS^
Diffuse	37.3% (19/51)	14 (40.00%)	5 (31.25%)	
Raynaud's Phenomenon				
Present	98.0% (50/51)	34 (97.14%)	16 (100.00%)	1.000^FE^
Absent	2.0% (1/51)	1 (2.86%)	0 (0.00%)	
Digital ulcer				
Present	68.6% (35/51)	32 (91.43%)	3 (18.75%)	<0.001^CS^
Absent	31.4% (16/51)	3 (8.57%)	13 (81.25%)	
Calcinosis				
Present	41.2% (21/51)	21 (60.00%)	0 (0.00%)	<0.001^CS^
Absent	58.8% (30/51)	14 (40.00%)	16 (100.00%)	
Vasodilator Use				
Yes	92.2% (47/51)	35 (100.00%)	12 (75.00%)	0.007^FE^
No	7.8% (4/51)	0 (0.00%)	4 (25.00%)	
Delta FTP				
< 40 mm	19.6% (10/51)	10 (28.57%)	0 (0.00%)	0.017^CS^
≥ 40 mm	80.4% (41/51)	25 (71.43%)	16 (100.00%)	
VAS pain				
> 70	17.6% (9/51)	7 (20.00%)	2 (12.50%)	0.514^CS^
≤ 70	82.4% (42/51)	28 (80.00%)	14 (87.50%)	

Data are

a Mean ± standard deviation.

b Median (1^st^ quartile ‒ 3^rd^ quartile or frequency of occurrence [number (%)]).

ST Student's *t* test.

MW *U* Mann Whitney test.

FE Fisher's Exact Test.

CS Chi-Square test; HAQ-DI, Health Assessment Questionnaire Disability Index; SHAQ, Scleroderma HAQ; CHFS, Cochin Hand Functional Scale; Delta FTP, Delta Finger-to-Palm.

### Main results

A predictive model for surgery indication

The multivariate binary logistic regression model identified the presence of DU as an independent predictor for the surgical indication (Table 2), increasing its chance by 46.2 times (OR_IC 95%_ = 8.23 to 259.49).

**Table 2:** Multivariate binary logistic regression for prediction of surgery indication.

Model	B	p-value	OR	CL 95%
Digital ulcers				
Absent			1.00	[Reference]
Present	3.83	< 0.001	46.22	8.23 to 259.49
Constant	−1.47	0.022	0.23	‒

Variables not in the equation: Health Assessment Questionnaire Disability Index (HAQ-DI:); Cochin Hand Functional Scale (CHFS); Delta Finger-to-palm (Delta FTP); Calcinosis; and Vasodilator Use. OR, Odds Ratio.

Other tested variables (HAQ-DI, CHFS, delta FT*P <* 40 mm, calcinosis, and vasodilator use) did not show an important contribution to the model for predicting the need for surgery in the presence of a DU.

Other analysis

**Fig. 1:**
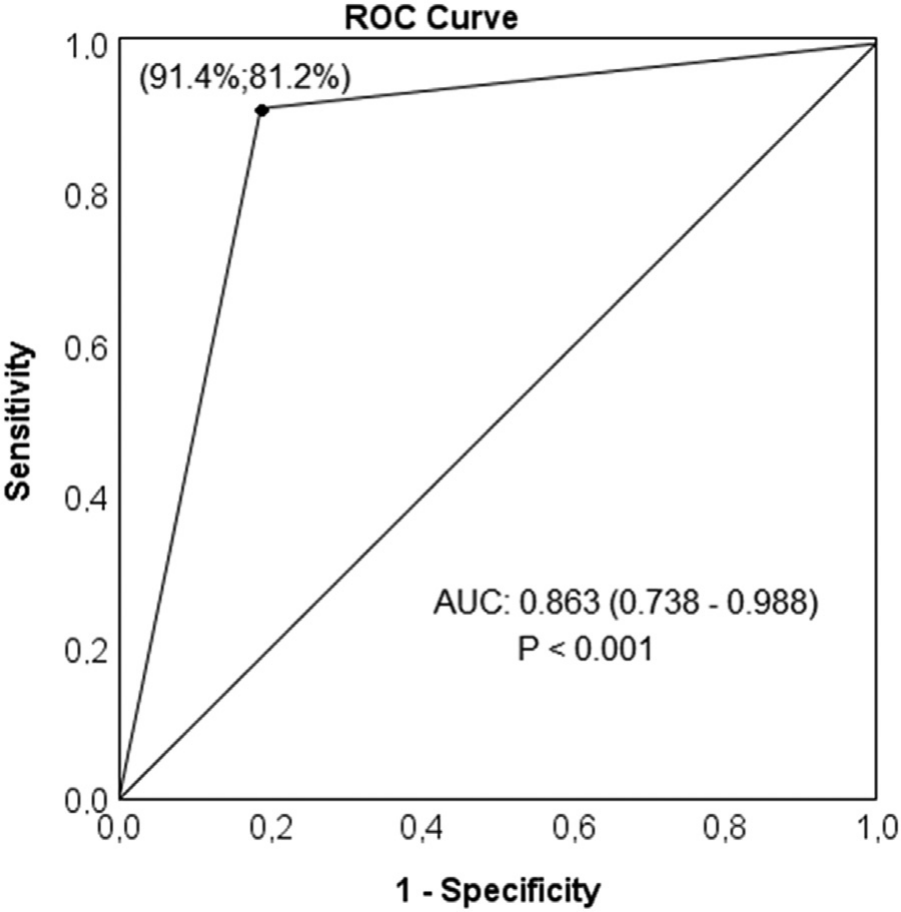
ROC curve analysis of values derived from the multivariate binary logistic regression model for prediction of surgical indication. The symbol “●” represents the sensitivity and specificity for surgery indication based on presence of digital ulcer. ROC, Receiver Operating Characteristic; AUC, Area Under the Curve.

This model based on the presence of DU demonstrated good accuracy (86.3%, *p <* 0.001) in predicting the need for hand surgical treatment in the studied population, with relevant values of sensitivity (91.4%) and specificity (81.2%) (Fig. 1).

## Discussion

### Synopsis of the key findings

Treatment of hand involvement in SSc can be uncomfortable and frustrating for patients and rheumatologists, especially when conservative treatment fails. In situations like this, a non-pharmacological approach is often necessary to enhance results. But often, the surgical option for the hands is sometimes underutilized as a treatment modality by rheumatologists. This can be explained by several factors, such as a lack of knowledge of rheumatologists regarding the existing surgical possibilities, inability to identify potential surgical candidates, inconsistent literature on possible surgical benefits, disagreement related to the ideal time for the referral and surgical efficacy, or even the lack of a reference hand surgeon to refer their patients.^8^^,^^23^

In addition to this, for various reasons, many professionals fail to practice more evidence-based medicine, sometimes guiding their practices mainly on personal experience. As a result, some patients do not receive the best treatment at the most appropriate time.^24^

Efforts to promote collaboration and referral between rheumatologists and hand surgeons to improve awareness of the possible advantages of operative management and to develop a more robust referral network could ensure that patients who may benefit from surgical treatment have an opportunity to be evaluated.

Also, early referral of such patients has the potential to a more effective management of the hand problem, lessen years of suffering and hand impairment of the patients, optimize resources in hand treatment, and may also result in better surgical outcomes due to less advanced disease.^23^^,^^25^

Previous studies have validated clinical signs and assessment tools to monitor SSc progression and assess the results of therapeutic interventions.^10^ However, to our knowledge, no study has used these monitoring tools to identify potential candidates for hand surgery.

In the present study, the authors hypothesized that one of these measures^26^ could be used for this purpose.

The idea was to use the findings to improve collaboration between the clinical and surgical teams, making surgical referrals earlier and faster.

The hypothesis was corroborated by multivariate analysis. A statistical association was found between the presence of DU and the eligibility for surgical treatment of the hand.

It was also found that potential surgical candidates had higher scores on the HAQ-DI and CHFS questionnaires, higher frequencies of calcinosis, use of vasodilators, and finger stiffness. However, no statistical association was found between these indicators and the expected outcome, perhaps a trend.

### Consideration of possible mechanisms and explanation

Patients with SSc have been experiencing higher survival rates in the last two decades because of the increase in knowledge about the disease and its treatment.^27^ As a result, the effects of disease-related disabilities have increased. In this context, hand manifestations are a well-known cause of disease-related disability, and their management is quite challenging.

One of the main characteristics of hand involvement in SSc is the presence of DU, which results from vasculopathy, cutaneous fibrosis, and joint contractures. It is present in 5% to 10% of patients, and about 50% of SSc patients will have a DU during their lifetime.^28^

The results of the multivariate analysis suggest that hands with DU respond more poorly to conservative treatment, which comes along with the fact that these patients have the disease for longer (mean 13.4 years vs. 6.4 years in our study), their hands are usually more deformed and have a worse function, especially when accompanied by pain.^29^^,^^30^

We believe that the results obtained could be extrapolated to other patients with SSc since the demographic characteristics of the studied group are comparable to those of other epidemiological studies done on SSc.^31^, ^32^, ^33^

### Limitations of the study

Our study is not without limitations. The results reported are from pilot observational research, not a randomized trial. As such, the clinical data collection was completed at the time of clinical care, which adds time limitations to the extent of data collected.

We also acknowledge that our patient population was limited, so the generalization of our findings should be taken with caution. However, this approach provides a basis to expand and confirm the results in a larger population of SSc patients.

Although we recognize that the clinical aspects of the disease influence the surgical treatment of the hand, our study limited itself to identifying potential candidates for hand surgery, regardless of their clinical condition.

### A brief section that summarizes the implications of the work for practice and research

The result of our study helps to shed some light on the matter of late surgical referrals for potential surgical candidates with SSc and hand problems, with the potential to assist in the interaction between the clinical and surgical teams, leading to more effective management and optimization of resources as part of a more evidence-based approach.

Therefore, the presence of DU in patients with SSc may be a screening tool to identify potential candidates for hand surgery, helping to enhance the collaboration between rheumatologists and hand surgeons as a part of a more evidence-based approach.

## Compliance with ethical standards

The following research was approved by the local Research Ethics Committee (protocol number: 23261013.8.0000.5404). All subjects received informed consent.

## Authors' contributions

Marcos Felipe Marcatto de Abreu: Data collection and Article writing.

Síbila Landin: Data collection.

Fernanda Yuri Yuamoto: Review the article.

Carolina Lins: Review the article

Eduardo Paiva Magalhães: Orientation.

Maurício Etchebehere: Orientation.

## Conflicts of interest

The authors M.F. Marcatto de Abreu, S. Landin, F.Y. Yuamoto, C. Lins, E. Paiva Magalhães, and M. Etchebehere declare that they have no conflict of interest.
